# Risk of breast cancer before and after rheumatoid arthritis, and the impact of hormonal factors

**DOI:** 10.1136/annrheumdis-2019-216756

**Published:** 2020-03-11

**Authors:** Hjalmar Wadström, Andreas Pettersson, Karin E Smedby, Johan Askling

**Affiliations:** 1 Clinical Epidemiology Division, Department of Medicine Solna, Karolinska Institutet, Stockholm, Sweden; 2 Patient Area Hematology, Theme Cancer, Karolinska University Hospital, Stockholm, Sweden; 3 Rheumatology, Theme Inflammation and Infection, Karolinska University Hospital, Solna, Sweden

**Keywords:** rheumatoid arthritis, epidemiology, early rheumatoid arthritis

## Abstract

**Objectives:**

To examine the risk of incident breast cancer in women with rheumatoid arthritis (RA), and the risk of RA in women with a history of breast cancer, taking antihormonal treatment for breast cancer into account.

**Methods:**

Using nationwide Swedish registers, women with new-onset RA diagnosed in 2006–2016 were identified and analysed using a cohort and a case–control design. Each patient with RA was matched on age, sex and place of residence to five randomly selected subjects from the general population. Through register linkages, we collected information on breast cancer, breast cancer risk factors (reproductive history and hormone replacement therapy) and socio-economy. The relative risk of breast cancer after RA was assessed using Cox regression, and the relative risk of RA in women with a history of breast cancer was assessed using conditional logistic regression.

**Results:**

The risk of incident breast cancer in women with RA was reduced and the association was not attenuated by adjustment for breast cancer risk factors (HR=0.80, 95% CI 0.68 to 0.93). The risk of RA in women with a history of breast cancer was similarly reduced (OR=0.87, 95% CI 0.79 to 0.95). Women with breast cancer treated with tamoxifen (OR=0.86, 95% CI 0.62 to 1.20) or aromatase inhibitors (OR=0.97, 95% CI 0.69 to 1.37) did not have an increased risk of RA compared with women with breast cancer treated differently.

**Conclusions:**

The decreased occurrence of breast cancer in patients with RA is present already before RA diagnosis; these reduced risks are not readily explained by hormonal risk factors. Adjuvant antihormonal therapy for breast cancer does not seem to increase RA risk.

**Key messages**

**What is already known about this subject?**

- The life-time risk of breast cancer is in the order of 1 woman out of 10. Several studies have reported a 15%–20% reduced risk of breast cancer in women with rheumatoid arthritis (RA), but the reasons behind this decrease remains unknown and unstudied.
- Previous studies have reported that tamoxifen and aromatase inhibitor treatment of breast cancer may increase the risk of RA.

**What does this study add?**

- We demonstrate that the risk of breast cancer after rheumatoid arthritis (RA) is indeed reduced, but this association cannot readily be explained by traditional breast cancer risk factors. By studying the future risk of RA in women with breast cancer, we demonstrate an equally pronounced risk reduction before RA diagnosis, again not explained by traditional breast cancer risk factors. These findings suggest that other factors, independent of RA, drive the inverse association between the two diseases.
- Tamoxifen and aromatase inhibitors do not seem to increase the risk of future RA.

**How might this impact on clinical practice or future developments?**

- With respect to patient counselling, the occurrence of a breast cancer in patients with rheumatoid arthritis (RA) is lower than in the general population, and tamoxifen and aromatase inhibitors appear safe in terms of risk of future RA.

## Background

With a lifetime risk of close to 1 in 10, breast cancer is the most common cancer among women. Rheumatoid arthritis (RA) is the most common rheumatic disease affecting the joints, and has a marked female predominance. Although the overall risk of malignancies in RA is increased by 10%–15% compared with the general population, large cohort studies have consistently reported decreased occurrence of breast cancer among women with RA.^1–3^

The characteristics and the reason(s) behind the decreased risk of breast cancer in women with RA remain unknown, indeed also virtually unstudied. Hormonal factors such as hormone replacement therapy (HRT),^4^ early menarche and late menopause,^5^ no breast feeding^6^ and nulliparity and increasing age at first birth^7^ are all established risk factors for breast cancer. The role of these factors in the occurrence of RA is less clear. For HRT, available evidence does not indicate an association with risk of RA.^8 9^ For breast feeding, studies to date have reported a protective effect against RA,^10–12^ while results for parity and risk of RA are inconclusive.^12–15^ Early menopause may be associated with subsequent development of RA.^10 16^ Importantly, however, no study has assessed the extent to which the decrease in risk of breast cancer in women with RA can be explained by known breast cancer risk factors.

If the observed reduction in the risk of breast cancer in women with RA was attributable to shared risk factors rather than a causal effect of the RA disease or its treatment, then one would expect that the reduced risk of breast cancer would be present already before the onset of RA. So far, the link between history of breast cancer and future risk of RA has been little investigated.^17^

The occurrence of RA following breast cancer is clinically and aetiologically important also for other reasons. Five to 10 years of adjuvant antihormonal treatment, for example, tamoxifen or aromatase inhibitors (AI), has become mainstay for oestrogen receptor positive breast cancer. Arthralgia is a common side effect of AI, and to a lesser extent also of tamoxifen. Some studies have suggested that these therapies not only induce arthralgia, but also inflammatory arthritis.^18 19^

Our study therefore had the following aims: first, to examine the risk of breast cancer in women with RA, and to investigate to what extent known breast cancer risk factors may explain the association. Second, to examine the risk of RA in women with a history of breast cancer, and whether antihormonal treatment for breast cancer modifies this association. To address the first aim, we used a cohort design, and to address the second aim, we used a case–control design, both applied to a nationwide population with new-onset RA and to individually matched population referents.

## Materials and methods

### Setting and data sources

Swedish healthcare is public and tax funded. Patients with RA are typically diagnosed and treated by rheumatologists. Sweden has national and virtually complete registers on demographics and health data which can be linked together by the unique personal identity number issued to all Swedish residents. This study was based on linkages between the Swedish Rheumatology Quality Register (SRQ), the National Patient Register (NPR), the Cancer Register, the Prescribed Drug Register, the Total Population Register, the Multigeneration Register and the Causes of Death Register, described elsewhere and in the online supplementary material.^20 21^

10.1136/annrheumdis-2019-216756.supp1 Supplementary data

## Study population and study design

Women with new-onset RA between 1 January 2006 and 31 December 2016 were identified from two partly overlapping sources, the SRQ and the NPR, using previously devised algorithms (online supplementary figure 1). Patients were required to have either (1) two visits in the NPR with an RA diagnosis (main or contributory) in outpatient specialist care, and at least one visit in a rheumatology or internal medicine clinic or (2) an RA diagnosis in SRQ. An index date was defined as the date of disease debut in the SRQ, RA diagnosis in the SRQ or first RA diagnosis in the NPR (inpatient or outpatient care, see online supplementary material for International Classification of Diseases (ICD) codes), whichever came first. Subjects who had not fulfilled the criteria (1 or 2) within 18 months of the index date were excluded. For each individual with RA, we randomly selected five population referents from the general population, matched on year of birth, sex and place of residence at the time of index date. For the cohort analysis of risk of breast cancer in women with RA, the population referents functioned as general population comparators. For the case–control analysis of risk of RA in women with a history of breast cancer, the population referents functioned as incidence-density sampled controls.

## Breast cancer and antihormonal treatment after breast cancer

Through linkage to the Swedish Cancer Register, all incident (1958 or later) cases of breast cancer (ICD 170, non-invasive and invasive) in the study population were identified. In the Prescribed Drug Register, we identified all dispensed prescriptions of tamoxifen and tamoxifen-like substances (99.6% tamoxifen, and hereafter referred to as tamoxifen) and AI, respectively, in July 2005 or later (see Anatomical Therapeutic Chemical (ATC) Classification System codes in online supplementary material).

## Covariates

We included information on educational level (from the Register of Total Population), country of birth, number of live births and age at first full-term pregnancy (from the Multigeneration Register), family history in a first-degree relative of breast cancer or ovarian cancer (via linkage to the Multigeneration and Cancer Registers), oral contraceptives and intrauterine devices and HRT (from the Prescribed Drug Register). Age 50 was used as a proxy for menopausal status. Status on all covariates were ascertained at index date. For details and for an assessment of the association between these covariates and the outcome please see online supplementary material.

SAS V.9.4 was used for all analyses. The study was approved by the Stockholm Ethics Review Board.

## Statistical analyses

To assess and characterise the risk of breast cancer in patients with RA, counting from RA diagnosis, we used a matched cohort design where patients with RA were considered exposed, and their population comparators unexposed. Start of follow-up for patients with RA (and for their matched population referents) was set to the date when all RA-defining inclusion criteria were fulfilled. Women with a history of breast cancer at the time of start of follow-up were excluded. End of follow-up was defined as 31 December 2016, death, emigration or breast cancer, whichever occurred first. Relative risks for breast cancer were assessed using Cox regression with follow-up time as the timescale. We first analysed age-adjusted models, and then gradually added more variables. In the full model, adjustments were made for age, calendar year, country of birth, educational level, HRT, oral contraceptives, age at first live birth, number of live-born children, family history of breast or ovarian cancer and previous invasive cancer. The risk of breast cancer was assessed overall, stratified by time since start of follow-up (<1 year, 1 to <5 years, >5 years), RA serostatus and age at RA diagnosis (18–49 years, 50–75 years and >75 years). Relative risks were also assessed separately for premenopausal and postmenopausal cancer, and tumour, node, metastases (TNM) cancer stage at diagnosis (online supplementary material).

To assess the relative risk of RA in women with a history of breast cancer, we used a case–control design, and calculated ORs using conditional logistic regression. Besides the matching variables (year of birth, sex and place of residence), the fully adjusted model accommodated country of birth, educational level, age at first live birth, number of live-born children and family history of breast or ovarian cancer. ORs were assessed overall, and stratified by age at RA diagnosis (18–49 years, 50–75 years, >75 years) and RA serostatus. Also, ORs were assessed according to different exposure definitions, that is, time between the breast cancer and RA (<1 year, 1 to <5 years, 5 to <10 years, >10 years), premenopausal or postmenopausal breast cancer and TNM cancer stage at diagnosis (see online supplementary material).

To assess the risk of RA following antihormonal breast cancer treatment, we again used a case–control design. Exposure was defined as at least two dispensings of the antihormonal drug in question, between breast cancer diagnosis and the index date. Also, analyses of cumulative exposure of antihormonal treatment were performed (<6 months, 6 to <12 months, 12 to <18 months, 18 to <24 months and ≥24 months), in these only one dispensing of antihormonal treatment was required. Since the Prescribed Drug Register started in July 2005, we restricted these analyses to women diagnosed with breast cancer from 2003 to 2016. As this would allow some patients receiving antihormonal treatment being misclassified as non-exposed, we performed a sensitivity analysis restricting the study period to July 2005 through December 2016. To avoid the inclusion of occult RA diagnosed shortly after breast cancer diagnosis, and misclassified RA due to acute arthralgia following initiation of antihormonal treatment, in a sensitivity analysis we excluded subjects with <1 year between the breast cancer diagnosis and index date.

## Results

### Characteristics of the study population

During 2006–2016, we identified 15 921 incident patients with RA, who were matched with 79 441 subjects from the general population (table 1). Mean age at index date was 59 years, and 68% were seropositive (5% unclassified, 6% had conflicting information).

**Table 1:** Characteristics of study population of Swedish women with RA 2006–2016, and matched population referents

	RA	Population referents
Persons (N)	15 921	79 441
Birth year (Q1–Q4)	1952 (1940–1963)	1952 (1940–1963)
Mean age at entry (SD)	59 (16)	59 (16)
Education (%)		
9 years or less	27	25
10–12 years	56	55
More than 12 years	15	19
RA in a first-degree relative (%)	10	4
Breast or ovarian cancer in a first-degree relative (%)	10	10
Invasive cancer prior to entry (%)	5	6
Country of birth (%)		
Sweden	84	83
Rest of Europe	11	11
Rest of the world	5	6
ACPA and RF negative	28	NA
ACPA or RF positive	68	NA
Mean age at first live birth (SD)	25 (5.0)	25 (5.0)
Children at index date (SD)	2 (1.4)	2 (1.4)
Combined oestrogen and progestin HRT (%)	5	5
Unopposed oestrogen HRT (%)	6	6
Combined oestrogen and progestin contraceptive (%)	9	9
Progestin only contraceptive (%)	7	7

ACPA, Anti-citrullinated protein antibodies; HRT, hormone replacement therapy; RA, rheumatoid arthritis; RF, Rheumatoid factor.

## Occurrence and relative risk of breast cancer in women with RA

During a mean follow-up of 5.00 years among patients with RA and 5.04 years among general population comparators, we identified 190 cases of breast cancer among 15 356 patients with RA, and 1191 cases among 75 854 population comparators, resulting in an age-adjusted and calendar year-adjusted HR=0.80 (95% CI 0.68 to 0.93). This estimate was virtually unchanged by further adjustments (fully adjusted HR=0.80, 95% CI 0.68 to 0.93). The risk was similar among seronegative RA (HR=0.77, 95% CI 0.58–1.02) and seropositive RA (HR=0.81, 95% CI 0.67–0.98), and for all age groups. We noted reduced risks for all TNM stages, and for both premenopausal and postmenopausal breast cancer (table 2).

**Table 2:** Relative risk of breast cancer in RA versus general population comparators (cohort study), adjusted for, age, country of birth, educational level, HRT, oral contraceptives, age at first birth, number of children, family history of breast cancer/ovarian cancer, previous invasive cancer and calendar year

	Patients with RA, Number	Population comparators, Number	HR (95% CI)
Overall	190	1191	0.80 (0.68 to 0.93)
Exposure variants			
RA, 18–49 years	18	155	0.59 (0.36 to 0.97)
RA, 50–75 years	146	841	0.87 (0.73 to 1.03)
RA, >75 years	26	195	0.68 (0.45 to 1.03)
RA 18–50 and breast cancer <50 years	9	53	0.86 (0.42 to 1.75)
Seronegative RA	55	346	0.77 (0.58 to 1.02)
Seropositive RA	124	772	0.81 (0.67 to 0.98)
Outcome variants			
Breast cancer, 18–50 years	9	102	0.47 (0.24 to 0.93)
Breast cancer, >50 years	181	1089	0.83 (0.71 to 0.97)
Stage 0	12	97	0.63 (0.34 to 1.14)
Stage 1	85	490	0.87 (0.69 to 1.09)
Stage 2	60	375	0.80 (0.61 to 1.05)
Stage 3	4	41	0.49 (0.17 to 1.36)
Stage 4	6	30	0.99 (0.41 to 2.39)

HRT, hormone replacement therapy; RA, rheumatoid arthritis.

## Occurrence and relative risk of RA in women with a history of breast cancer

At index date, 555 (3.5%) of the 15 921 patients with RA, and 3193 (4.0%) of the controls had a history of breast cancer, age-adjusted OR=0.86 (95% CI 0.78 to 0.94), fully adjusted OR=0.87 (95% CI 0.79 to 0.95, table 3). ORs stratified by serostatus and age at RA diagnosis yielded similar results. There was no clear trend when examining the risk by menopausal status, or cancer stage, at breast cancer diagnosis. However, missing information on cancer stage was substantial, especially among earlier cases of cancer.

**Table 3:** Relative risk of RA in women with a history of breast cancer (case–control study), adjusted for, age, country of birth, educational level, age at first birth, number of children and family history of breast cancer/ovarian cancer

	RA (n)	Controls (n)	OR (95% CI)
Overall	555	3193	0.87 (0.79 to 0.95)
Exposure variants			
Breast cancer, 18–50 years	123	761	0.82 (0.67 to 0.99)
Breast cancer, >50 years	432	2432	0.89 (0.80 to 0.98)
Stage 0, 2002–2016	28	130	1.10 (0.73 to 1.65)
Stage 1, 2002–2016	110	530	1.04 (0.84 to 1.27)
Stage 2, 2002–2016	61	456	0.67 (0.51 to 0.87)
Stage 3, 2002–2016	9	33	1.35 (0.65 to 2.83)
Stage 4, 2002–2016	1	17	0.29 (0.04 to 2.19)
Outcome variants			
RA, 18–49 years	24	111	1.09 (0.70 to 1.71)
RA, 50–75 years	357	2132	0.84 (0.75 to 0.94)
RA, >75 years	174	950	0.91 (0.77 to 1.08)
Breast cancer 18–50 and RA >50 years	99	650	0.77 (0.62 to 0.96)
Seronegative RA	157	921	0.85 (0.71 to 1.01)
Seropositive RA	367	2088	0.88 (0.78 to 0.98)

RA, rheumatoid arthritis.

## Relative risk of breast cancer by time before and after RA

**Figure 1:**
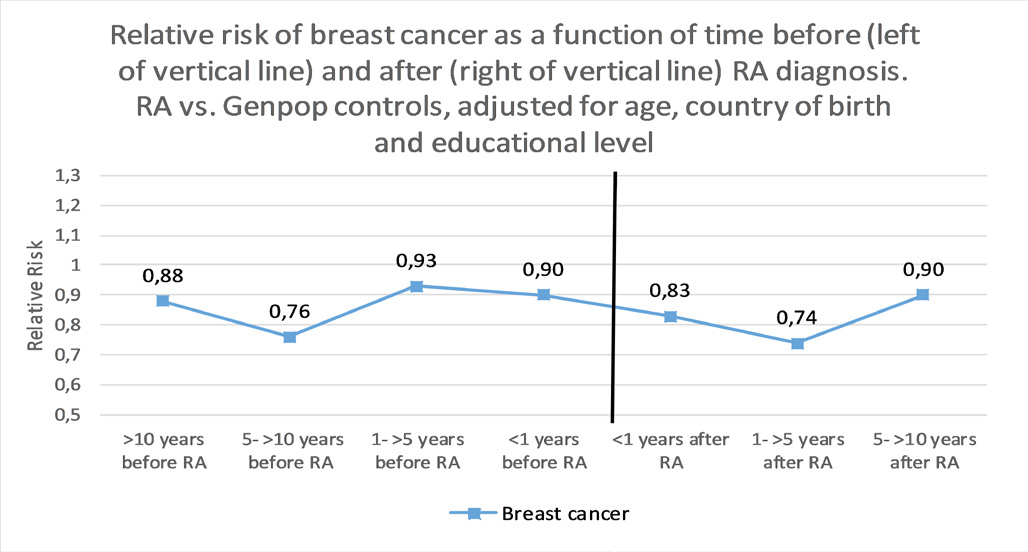
Relative risk of breast cancer (invasive or in situ) as a function of time before (left of vertical line, ORs) and after (right of vertical line, HRs) RA diagnosis. RA versus general population comparators, adjusted for age, country of birth and educational level. RA, rheumatoid arthritis

There was no clear trend when stratifying by follow-up time (figure 1). The lowest OR for breast cancer was observed 5 to <10 years before RA, OR=0.76 (95% CI 0.63 to 0.93), and the highest 1 to <5 years before RA, OR=0.93 (95% CI 0.58 to 1.38). The lowest HR for breast cancer was observed 1 to <5 years after RA, HR=0.74 (95% CI 0.60 to 0.91), and the highest 5 to <10 years after RA, HR=0.90 (95% CI 0.67 to 1.20).

## Occurrence and relative risk of RA following anti-hormonal treatment among women with a history of breast cancer

A history of a first ever breast cancer occurring during 2003–2016 was identified among 259 of the 15 921 patients with RA, and 1499 of the 79 441 controls (table 4). Among these subjects, 117 (45%) patients with RA and 631 (42%) controls had not received tamoxifen or AI treatment before RA diagnosis/index date (never vs ever, fully adjusted OR=1.23, 95% CI 0.92 to 1.64).

**Table 4:** Relative risk of RA in women, with or without a history of breast cancer, by antihormonal treatment before RA diagnosis, adjusted for age, education, age at first birth, number of children and family history of breast cancer/ovarian cancer

	RA (N)	Controls (N)	Fully adjusted OR (95% CI)
No breast cancer	15 366	76 248	1.07 (0.88 to 1.31)
Never AI or tamoxifen	117	631	REF
Only tamoxifen versus never	62	383	0.86 (0.62 to 1.20)
Only AI versus never	59	322	0.97 (0.69 to 1.37)
Both AI and tamoxifen versus never	21	163	0.68 (0.41 to 1.12)

AIs, aromatase inhibitors; RA, rheumatoid arthritis.

Among the remaining 1010 patients with RA and controls, use of tamoxifen was somewhat more frequent than AI (n=629 vs n=565), with some overlap (n=184). The risk of RA was not significantly associated with any combination of antihormonal breast cancer treatments; both tamoxifen and AI versus never treated: fully adjusted OR=0.68 (95% CI 0.41 to 1.12); tamoxifen-only versus never treated: OR=0.86 (95% CI 0.62 to 1.20); AI-only versus never treated: OR=0.97 (95% CI 0.69 to 1.37; table 4).

When examining cumulative tamoxifen exposure (years), we noted no trends in risk of RA (table 5). For AI, we noted an increased risk of RA among patients treated with AI for <6 months (OR=1.58, 95% CI 1.02 to 2.45), but also a decreased risk with longer exposure time of >24 months OR=0.57, 95% CI 0.39 to 0.82).

**Table 5:** Relative risk of RA in patients with a history of breast cancer, by accumulated time of antihormonal treatment before RA diagnosis, adjusted for age, education, age at first birth, number of children and family history of breast cancer/ovarian cancer

Cumulative exposure	Tamoxifen			AIs		
	RA (n)	Controls (n)	OR (95% CI)	RA (n)	Controls (n)	OR (95% CI)
<6 months	27	134	0.98 (0.65 to 1.49)	27	87	1.58 (1.02 to 2.45)
6 to <12 months	14	76	0.95 (0.53 to 1.68)	9	63	0.72 (0.36 to 1.44)
12 to <18 months	8	62	0.62 (0.30 to 1.30)	12	41	1.45 (0.76 to 2.77)
18 to <24 months	11	65	0.83 (0.43 to 1.57)	12	57	1.08 (0.58 to 2.02)
>24 months	42	283	0.74 (0.54 to 1.03)	31	267	0.57 (0.39 to 0.82)

AIs, aromatase inhibitors; RA, rheumatoid arthritis.

Sensitivity analyses restricting the analysis of risk of RA among women with a history of breast cancer to cancer cases occurring after the Prescribed Drug Register was started (July 2005 to December 2016), and excluding subjects with <1 year between the breast cancer diagnosis and index date, provided results similar to the main analysis (see online supplementary material).

## Discussion

In this large population-based study, we made a series of important observations. Also in this recent modern cohort of patients followed from RA diagnosis, there was a decreased risk of breast cancer, corresponding to a lifetime risk of 1 in 12.5, instead of 1 in 10 as in the general population.^22^ This risk reduction was independent of RA serostatus, and remained after adjusting for breast cancer risk factors. Furthermore, the risk of future RA in women with a history of breast cancer was reduced with risk estimates similar to those of breast cancer risk after RA. Finally, treatment of breast cancer with tamoxifen and AI did not constitute risk factors for the development of RA.

We noted, as has previously been reported,^1^ a 20% decreased risk of breast cancer in women with a history of RA, and extend this finding by demonstrating that adjusting for several important breast cancer risk determinants did not significantly impact this result. The relative risk of RA in women with a history of breast cancer was similar to the relative risk of breast cancer in women with a history of RA. Taken together, this would argue against the hypothesis that the decreased risk of breast cancer in patients with RA is due to the RA disease or its treatment. Rather, our data suggest that RA and breast cancer share genetic factors or environmental factors acting earlier in life. In this regard, it is interesting that the breast cancer ORs and HRs were similar for seropositive and seronegative RA.

This is one of the first studies examining the relationship between antihormonal breast cancer treatment, and the risk of RA.^18 19^ Contrary to a study by Caprioli *et al*, who studied the risk of RA in a cohort of 10 493 women with breast cancer, we did not find a markedly higher risk of RA in AI-treated compared with tamoxifen-treated patients. Although based on relatively small numbers, our upper confidence limits indicate a low probability for clinically significant risk increases overall. However, we cannot rule out an association between RA and tamoxifen/AI among specific subgroups, for example, as defined by hormonal receptor status, which we did not have information on. We did observe a trend towards a higher risk in the beginning of treatment, but then a reduced risk after 2 years of AI treatment. Caprioli *et al* did not compare the rate of RA among patients with breast cancer to that of the general population. The rate that they observed (4.33 per 1000 person-years) is somewhat lower than population-based age and sex standardised incidence rates reported from Italy^23^ and Sweden.^24^ Our results also differed from that of a cross-sectional study by Chen *et al* that included more than 200 000 cases of breast cancer.^18^ They found a cumulative dose-dependent risk increase of RA with both tamoxifen and AI treatment, compared with women with breast cancer who did not receive these treatments. However, they neither took follow-up time nor age into account. We believe that our nationwide study with a clearly defined population, which could account for person-time at risk, age and several other potential confounders, and the use of an appropriate comparator group, provides a more reliable estimate.

We adjusted our models for hormonal risk factors, which had little impact on our results. Whether this is due to residual confounding or a true absence of confounding is difficult to ascertain, but we did observe that these factors were indeed risk factors for breast cancer in our study population (online supplementary material). Studies have found that certain polymorphisms of the cyclooxygenase-2 gene promoter are associated with an increased risk of breast cancer and a decreased risk of RA.^25 26^ Also, polymorphisms in the DRB1 gene, the major genetic susceptibility locus for RA,^27^ have recently been linked to a decreased risk of breast cancer.^28^ However, because of the complex inheritance, it is difficult to estimate the net effect of such singular genetic factors.

Our study has some limitations. Although algorithms for identifying incident RA diagnoses in the NPR have been validated and shown to have a positive predictive value close to 90%, misclassification of RA cannot be excluded.^29^ We lacked data on menarche, menopause and breast feeding. Early menarche has been reported as a risk factor, and long-term breast feeding seems protective, for both RA and breast cancer.^10–12^ Early menopause, which is negatively associated with breast cancer, has been reported as a risk factor for RA.^11 16^ We also could not account for some additional potential confounding factors including body mass index, smoking and alcohol consumption. Body mass index is considered a risk factor for both breast cancer and RA and would thus introduce a positive confounding.^30^ Smoking is a risk factor for RA, but alcohol has been reported as being protective against RA.^31^ However, both are only weak risk factors for breast cancer.^32 33^ We were not able to account for mammographic screening. However, the negative association between RA and breast cancer was described in studies conducted before mammographic screening was mainstay.^34 35^ Moreover, since the assessment of RA risk after breast cancer is, by definition, an assessment among breast cancer survivors, we cannot exclude that breast cancer among would-be patients with RA could have a worse prognosis irrespective of cancer stage.^36 37^

The strengths of this study were its large sample size and long follow-up, the use of nationwide registers with high internal validity and coverage and that we could adjust our models for several potentially important confounders.

In conclusion, we found a decreased risk of breast cancer in patients with RA, and a similar decrease in risk of RA in patients with a history of breast cancer. We did not find evidence to support that the decreased risk of breast cancer was due to known risk determinants. Thus we were ultimately unable to explain the origins of this association. Antihormonal therapy as used in secondary breast cancer pharmacoprevention does not seem to increase RA risk.
