# Supplementary material for: Risk of breast cancer before and after rheumatoid arthritis, and the impact of hormonal factors
Source: Ann Rheum Dis. 2020 Mar 11;79(5):581–6. doi: 10.1136/annrheumdis-2019-216756 (PMC7213316; doi:10.1136/annrheumdis-2019-216756)
Supplement: Supplementary data [file annrheumdis-2019-216756supp001.pdf]

Supplementary materials

Additional information about the data sources

The Swedish Rheumatology Quality Register is a nationwide, profession based register started in 1996. The National Swedish Patient Register is a nationwide register listing diagnosis, dates, hospital and department. Diagnoses are listed as assigned by the discharging physician, and coded according to ICD-10. The inpatient subset has near complete coverage since 1987, the outpatient subset since 2001. A similar algorithm to the one we used for identifying incident RA patients in the Patient Register has been validated (1). Among individuals identified with incident RA using an algorithm for new-onset disease, the RA diagnosis was substantiated in 91%, of whom 92% also represented new-onset disease. The Swedish Cancer Register was established in 1958, reporting incident cancers is mandatory for both clinicians and pathologists/cytologists and coverage is estimated to be greater than 95% (2). The register contains data on date of diagnosis, and type of cancer according to the ICD classification and morphology/histology, as well as TNM-stage (since 2002). ICD7 is the coding system used when the Cancer register was started in 1958. Since then, newer versions of ICD have been implemented in parallel so that new cancers are currently coded according to ICD10, and also according to previous ICD-versions which facilitates comparisons over time. The Swedish Prescribed Drug Register contains information on all dispensations of prescribed drugs in Sweden since July 2005. It lists the article, quantity, dose, date, and the personal identification number of the patient. The Total Population Register lists data on residency at a given point in time ever since it was founded in 1961, including dates of emigration/immigration for all subjects ever resident in Sweden since 1961. The Multi-generation register, which is part of the Total Population Register, allows for linkage of individuals to parents, children, and siblings. The Causes of Death Register is a national register that provides date and causes of death for all deceased residents since 1961.

Additional information about covariates

Country of birth was categorized as Sweden, Europe excluding Sweden, the rest of the World excluding Europe. Family history of breast cancer or ovarian cancer in a first-degree relative, was identified and dicotomized (yes/no). Number of live births was categorized as 0, 1, 2, 3 or more, and assessed at index date, births occuring after a breast cancer diagnosis were not considered. Age at first full-term pregnancy categorized as <26 years, 26 years or older, and no children, again births occuring after a breast cancer diagnosis were not considered. Using the PDR, all oral contraceptives and intrauterine devices 2006- was identified and categorized as combined estrogen and progestin, or progestin only. All hormone replacement therapy 2006-, respectively, was identified and categorized as combined estrogen and progestin, or estrogen only (see ATC-codes above).

Additional information on methods behind defining the study population

Patients with disease debut before Jan 2006 or before their 18th birthday were excluded. Patients with a diagnosis of systemic lupus erythematous (SLE) in the NPR prior to the index date were excluded in analyses of risk of RA in women with a history of breast cancer. In analyses of risk of breast cancer in women with a history of RA subjects with an SLE diagnosis before index were excluded, and subjects were also censored at first SLE diagnosis during follow-up. Anti–citrullinated protein antibody- (ACPA) and rheumatoid factor status was

captured through both SRQ and NPR and defined as seropositive (RF or ACPA positive), seronegative (RF and ACPA negative), or unknown. If present, serostatsus in SRQ took precedence. If serostatus was not recorded in SRQ, patients with at least one rheumatoid factor positive, or ACPA positive, RA diagnosis in NPR were considered seropositive. Patients with at least one seronegative diagnosis in NPR and no seropositive diagnosis were considered seronegative. Serostatus for the remaining patients was classified as unknown.

List of ATC codes

Aromatase inhibitors

L02BG01 aminoglutethimide

L02BG02 formestane

L02BG03 anastrozole

L02BG04 letrozole

L02BG05 vorozole

L02BG06 exemestane

Anti estrogens

L02BA01 tamoxifen

L02BA02 toremifene

L02BA03 fulvestrant

Hormone replacement therapy

G03CA01 etinylestradiol

G03CA03 estradiol

G03CA04 estriol

G03CA57 conjugated estrogens

G03CC05 dietylstilbestrole

G03CX01 tibolone

G03DA02 medroxiprogesterone

G03DA04 progesterone

G03DB01 dydrogesterone

G03DB08 dienogest

G03DC02 noretisterone

G03DC03 lynestrenole

G03FA01 norethisterone and estrogen

G03FA12 medroxyprogesterone and estrogen

G03FA15 dienogest and estrogen

G03FA17 drospirenone and estrogen

G03FB05 norethisterone and estrogen

G03FB06 medroxyprogesterone and estrogen

G03FB09 levonorgestrel and estrogen

Contraceptives

G02BA03 plastic IUD with progestogen

G03AA03 lynestrenol and ethinylestradiol

G03AA05 norethisterone and ethinylestradiol

G03AA07 levonorgestrel and ethinylestradiol

G03AA09 desogestrel and ethinylestradiol

G03AA11 norgestimate and ethinylestradiol

G03AA12 drospirenone and ethinylestradiol

G03AA13 norelgestromin and ethinylestradiol

G03AA14 nomegestrol and estradiol

G03AB02 lynestrenol and ethinylestradiol

G03AB03 levonorgestrel and ethinylestradiol

G03AB04 norethisterone and ethinylestradiol

G03AB05 desogestrel and ethinylestradiol

G03AB08 dienogest and estradiol

G03AC01 norethisterone

G03AC02 lynestrenol

G03AC03 levonorgestrel

G03AC06 medroxyprogesterone

G03AC08 etonogestrel

G03AC09 desogestrel

List of ICD codes

RA ICD 10: M05, M06

Other arthritis diagnoses ICD 10: M07, M08, M09, M45, M13, M12, M468

SLE ICD 10: M32

Breast Cancer ICD 7:170, ICD 9:174, ICD 10: C50

Ovarian Cancer ICD 7:175 ICD 9:183, ICD 10: C56, C57.0

TNM Stage

Mx was classified as M0

Nx was excluded in analysis stratified on clinical stage

Stage 0
Tis N0 M0

Stage I
T1 N0 M0

Stage II
T2-3 N0 M0
T1-2 N1 M0

Stage III
T3 N1 M0
T1-3 N2 M0
T4 N0-2 M0
T1-4 N3 M0

Stage IV
T1-4 N0-3 M1

Supplementary Table 1. Strength of association between potential confounders and the outcome (any breast cancer) among the population comparators, age-adjusted.

	HR (95% CI)
Education	
missing	0.36 (0.15-0.88)
9 years or less	REF
10-12 years	1.13 (0.98-1.30)
more than 12 years	1.28 (1.07-1.53)
Breast- or ovarian cancer in a first degree relative,	
YES	1.78 (1.52-2.08)
NO	REF
Invasive cancer prior to entry %	
YES	0.93 (0.72-1.20)
NO	REF
Country of birth	
Sweden	REF
Europe excluding Sweden	0.94 (0.78-1.13)
The World, excluding Europe	0.61 (0.42-0.87)
Missing	NA (only 9 individuals)
HRT	
Combined estrogen and progestin	2.22 (1.82-2.69)
Unopposed estrogen	1.43 (1.15 -1.77)
No HRT	REF
Hormonal contraceptives	
Progestin only contraceptive %	0.77 (0.56-1.06)
Combined estrogen and progestin contraceptive %	0.73 (0.54-0.95)
No hormonal contraceptive	REF
Age at first childbirth	
No children	REF
Younger than 26 years at first birth	1.00 (0.85-1.19)
26 years or older at first birth	1.17 (0.98-1.39)
Number of children	
0 children	REF
1 child	1.00 (0.82-1.23)
2 children	1.02 (0.85-1.21)
3 or more children	0.87 (0.72-1.05)

Supplementary table 2. Relative risk of developing RA in patients with a history of breast cancer, by accumulated time of antihormonal treatment before RA diagnosis (July 2005-Dec 2016) adjusted for age, education, age at first birth, number of children, family history of breast cancer/ovarian cancer

	Tamoxifen			Aromatse Inhibitors		
Cumulative exposure	RA n	Controls n	OR (95% CI)	RA n	Controls n	OR (95% CI)
<6 months	23	108	1.06 (0.67-1.66)	22	69	1.63 (1.00-2.66)
6-<12 months	10	54	0.96 (0.49-1.89)	9	54	0.83 (0.41-1.68)
12-<18 months	3	43	0.33 (0.10-1.07)	12	33	1.80 (0.93-3.48)
18-<24 months	6	44	0.64 (0.27-1.52)	6	43	0.72 (0.31-1.71)
>24 months	32	185	0.87 (0.60-1.27)	23	173	0.64 (0.41-0.99)

Supplementary table 3. Relative risk of developing RA in patients with a history of breast cancer, by antihormonal treatment before RA diagnosis (July 2005-Dec 2016) adjusted for age, education, age at first birth, number of children, family history of breast cancer/ovarian cancer

	RA n	Controls n	Fully adjusted OR (95% CI)
No breast cancer	15,366	76,248	1.16 (0.91-1.48)
Never AI or Tamoxifen	79	459	REF
Only Tamoxifen vs never	45	267	0.97 (0.65-1.45)
Only AI vs never	51	239	1.22 (0.83-1.80)
Both AI and Tamoxifen vs never	12	107	0.64 (0.34-1.22)

AI=Aromatase inhibitors

Supplementary table 4. Relative risk of developing RA in women with a history of breast cancer, by antihormonal treatment before RA diagnosis adjusted for age, education, age at first birth, number of children, family history of breast cancer/ovarian cancer. Patients with less than 1 year between breast cancer and index date excluded.

	RA n	Controls n	Fully adjusted OR (95% CI)
No breast cancer	15,165	74,945	1.03 (0.83-1.29)
Never AI or Tamoxifen	96	491	REF
Only Tamoxifen vs never	57	353	0.83 (0.58-1.19)
Only AI vs never	53	304	0.88 (0.61-1.26)
Both AI and Tamoxifen vs never	21	162	0.66 (0.40-1.09)

AI=Aromatase inhibitors

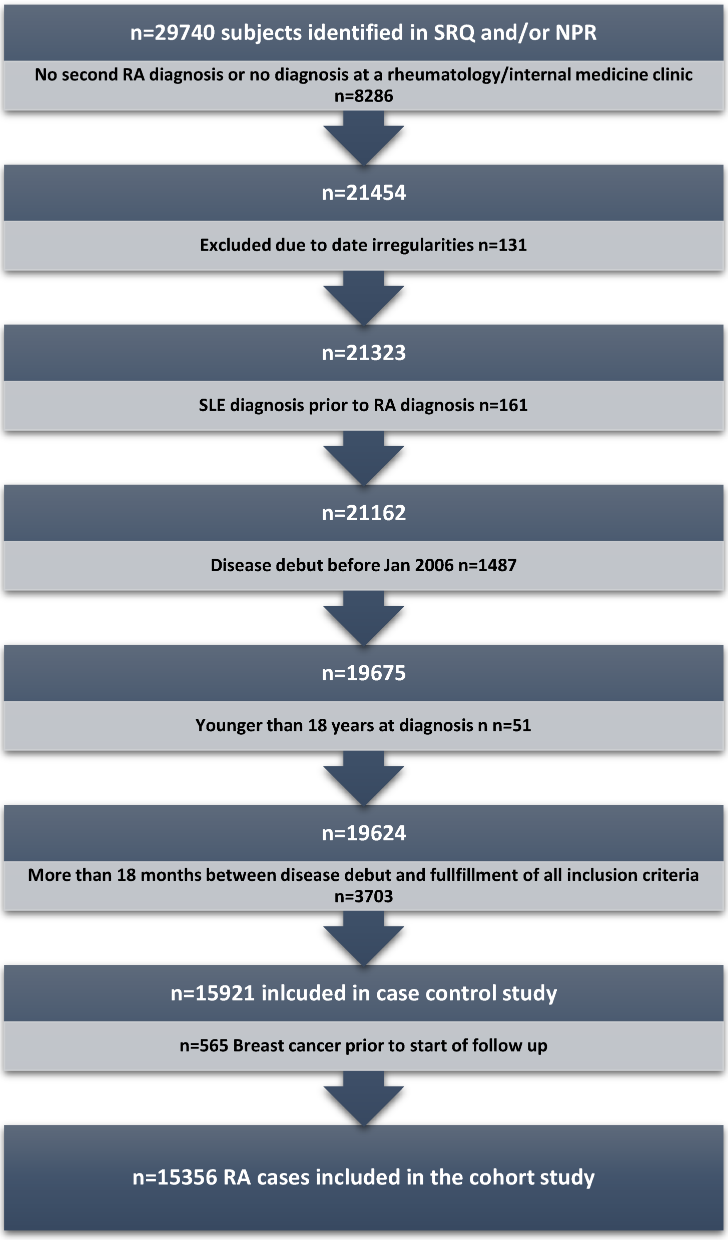

Supplementary Figure 1. Flowchart describing the process behind defining the study population.

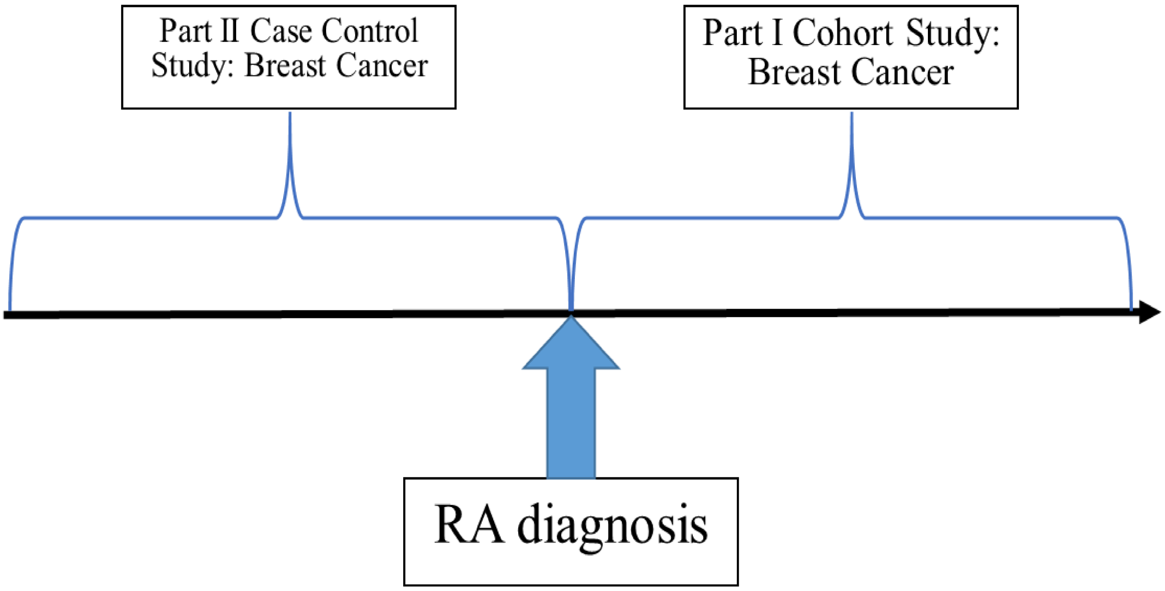

Supplementary Figure 2. Graphical description of part I, risk of breast cancer in women with a history of RA, and part II, risk of RA in women with a history of breast cancer.

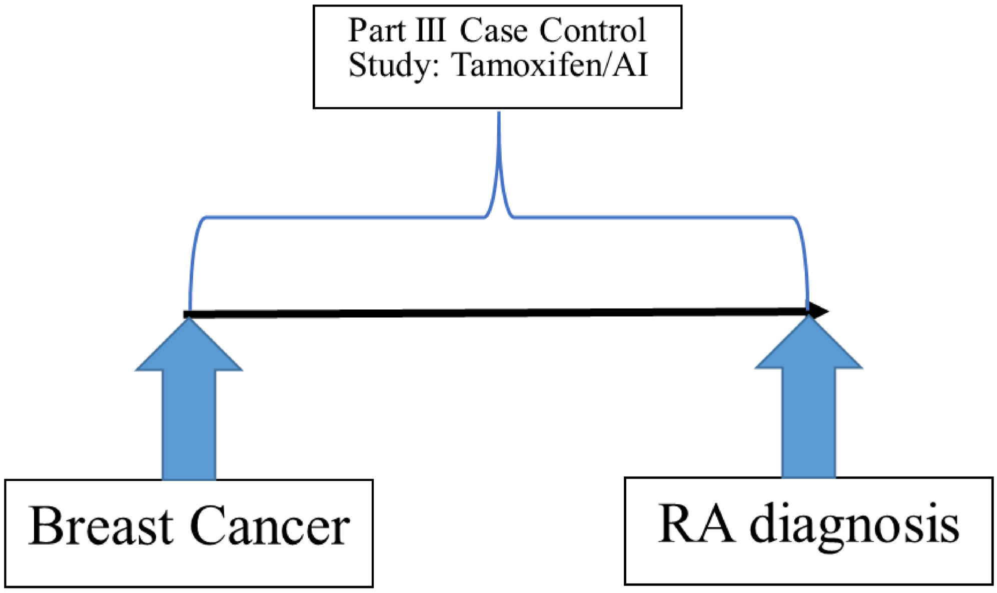

Supplementary Figure 3. Graphical description of part III, risk of RA following antihormonal treatment among women with a history of breast

References

1. Waldenlind K, Eriksson JK, Grewin B, Askling J. Validation of the rheumatoid arthritis diagnosis in the Swedish National Patient Register: a cohort study from Stockholm County. BMC musculoskeletal disorders. 2014;15(1):432.
2. Barlow L, Westergren K, Holmberg L, Talbäck M. The completeness of the Swedish Cancer Register – a sample survey for year 1998. Acta Oncologica. 2009;48(1):27-33.
